# TGF-β/Smad2/3 Signaling Directly Regulates Several miRNAs in Mouse ES Cells and Early Embryos

**DOI:** 10.1371/journal.pone.0055186

**Published:** 2013-01-30

**Authors:** Nicholas Redshaw, Carme Camps, Vikas Sharma, Mehdi Motallebipour, Marcela Guzman-Ayala, Spyros Oikonomopoulos, Efstathia Thymiakou, Jiannis Ragoussis, Vasso Episkopou

**Affiliations:** 1 Department of Medicine, Division of Brain Sciences, Imperial College London, London, United Kingdom; 2 Wellcome Trust Centre for Human Genetics, University of Oxford, Oxford, United Kingdom; Baylor College of Medicine, United States of America

## Abstract

The Transforming Growth Factor-β (TGF-β) signaling pathway is one of the major pathways essential for normal embryonic development and tissue homeostasis, with anti-tumor but also pro-metastatic properties in cancer. This pathway directly regulates several target genes that mediate its downstream functions, however very few microRNAs (miRNAs) have been identified as targets. miRNAs are modulators of gene expression with essential roles in development and a clear association with diseases including cancer. Little is known about the transcriptional regulation of the primary transcripts (pri-miRNA, pri-miR) from which several mature miRNAs are often derived. Here we present the identification of miRNAs regulated by TGF-β signaling in mouse embryonic stem (ES) cells and early embryos. We used an inducible ES cell system to maintain high levels of the TGF-β activated/phosphorylated Smad2/3 effectors, which are the transcription factors of the pathway, and a specific inhibitor that blocks their activation. By performing short RNA deep-sequencing after 12 hours Smad2/3 activation and after 16 hours inhibition, we generated a database of responsive miRNAs. Promoter/enhancer analysis of a subset of these miRNAs revealed that the transcription of pri-miR-181c/d and the pri-miR-341∼3072 cluster were found to depend on activated Smad2/3. Several of these miRNAs are expressed in early mouse embryos, when the pathway is known to play an essential role. Treatment of embryos with TGF-β inhibitor caused a reduction of their levels confirming that they are targets of this pathway *in vivo*. Furthermore, we showed that pri-miR-341∼3072 transcription also depends on FoxH1, a known Smad2/3 transcription partner during early development. Together, our data show that miRNAs are regulated directly by the TGF-β/Smad2/3 pathway in ES cells and early embryos. As somatic abnormalities in functions known to be regulated by the TGF-β/Smad2/3 pathway underlie tumor suppression and metastasis, this research also provides a resource for miRNAs involved in cancer.

## Introduction

The Transforming Growth Factor-β (TGF-β) superfamily comprises a broad range of signaling ligands including TGF-β, Activin, Nodal and Bone Morphogenetic Protein (BMP) [1]. These are essential for embryonic development and have been shown to regulate a variety of cellular processes such as migration, differentiation, proliferation and apoptosis [2], [3]. During embryonic development, these factors exhibit dose-dependent effects that provide positional information to cells and specify embryonic axes [4], [5]. They are essential for gastrulation, a process that depends on epithelial-to-mesenchymal transition (EMT) and the opposite (MET), and for the induction of mesendoderm and endoderm [4], [6], [7]. In adult tissues, TGF-β signaling has an important role in tissue homeostasis, and its misregulation often leads to a variety of diseases, most notably cancer [6], [8].

Binding of TGF-β, Activin and Nodal ligands to pairs of membrane-bound receptor serine/threonine kinases (type I and II receptors), results in the specific C-terminal phosphorylation of Smad2 and Smad3 (p-Smad2/3) in the cytoplasm. P-Smad2/3 then form heteromeric complexes with Smad4 and translocate to the nucleus where they directly regulate target gene expression [3], [9], [10]. Target gene specificity is assisted by a variety of Smad2/3 co-regulatory factors that confer tissue-specific gene expression. One important co-factor during early murine embryogenesis is the winged-helix transcription factor FoxH1, which interacts with p-Smad2/3 to activate target gene expression in response to Nodal signaling [11], [12], [13], [14], [15]. Smads regulate hundreds of target genes, of which a small number have been characterized as direct targets of the pathway [16], [17].

miRNAs have recently emerged as major regulators of gene expression [18], [19], [20]. They are first transcribed by RNA polymerase II as long primary transcripts, termed primary (pri-) miRNAs. Pri-miRNAs are subsequently processed by the RNase III endonuclease Drosha into ∼70 nt hairpin-shaped precursor (pre-) miRNAs. The pre-miRNA is then exported from the nucleus to the cytoplasm where it undergoes further processing to a ∼22 nt miRNA/miRNA* duplex by the Dicer enzyme complex [21], [22], [23], [24]. This duplex is unwound to release the mature miRNA (the miRNA* strand is typically degraded), which is finally loaded into the RNA-Induced Silencing Complex (RISC) where it mediates silencing of target mRNA via interactions with complementary regions within the 3′-UTR. When bound to their targets, miRNAs can promote mRNA degradation and/or block translation [20], [25]. miRNAs are located either within the introns of other genes or in intergenic regions, and are transcribed by the host gene or by their own promoter [20]. Such transcripts of pri-miRNA may contain clusters that are processed to several different mature miRNAs. However, little is known about the regulation of pri-miRNA promoters [26], [27], [28].

Thousands of miRNAs have now been identified in a range of organisms [29], and many of their targets have been revealed [30]. Studies on the deregulation of miRNA processing provide strong evidence that their function in fine-tuning gene expression is essential in the development of almost every tissue and body system examined [31], [32], [33], [34], [35]. Furthermore, miRNA deficiencies and excesses have been correlated with a number of clinically important diseases, including cancer [36], [37], [38].

Deletion of the Dicer gene in mice results in embryonic lethality and a depletion of the stem cell population [32]. Conditional tissue-specific inactivation of Dicer mutations confirm the essential role of miRNAs throughout development [31], [32], [34,]. More recent studies implicate specific miRNAs and their targets in early development [39], [40], [41], [42], [43]. For example, Martello and colleagues demonstrated that miRs-15 and -16 regulate embryonic patterning in *Xenopus* as they restrict the size of the Spemann’s organizer by targeting the Nodal type II receptor, Acvr2a [41]. In mouse models, studies on the miR-290∼295 cluster revealed a crucial role in pluripotency and differentiation [39], [40], [42], [43]. It is highly abundant in undifferentiated mouse ES cells and decreases with differentiation [40]. Consistent with this, members of the miR-290∼295 cluster have been shown to promote the G1-S transition and thereby promote the rapid proliferation of mouse ES cells [43].

A number of studies have demonstrated that Smad2/3 participate in the post-transcriptional regulation of miRNAs [44], [45], [46]. This mechanism involves the binding of Smads to Smad Binding Elements (SBE) in the stem region of pri-miRNAs. This subsequently promotes pri- to pre-miRNA processing by facilitating Drosha- and DGCR8-mediated cleavage [44], [45]. However, accumulating evidence now demonstrates that miRNA genes are also transcriptionally regulated by Smads. SMAD3 and SMAD4 have been shown to directly regulate miRs-143 and -145 in human coronary artery smooth muscle cells [47]. In gastric cancer cells, SMAD3 has a demonstrated role in directly regulating miR-200 family members, which subsequently target ZEB1 and ZEB2, which are both repressors of E-cadherin transcription, and therefore involved in EMT [48]. Despite the importance of the Nodal-Smad2/3 branch of TGF-β signaling in regulating ES cell pluripotency, differentiation and patterning in the early mouse and other vertebrate embryos [49], [50], [51], [52], [53], a systematic screen for miRNAs regulated by such signals has, to our knowledge, not been performed.

In this study, we identify miRNA targets of the TGF-β/Smad2/3 pathway in mouse ES cells by deep-sequencing. We used a previously developed system in ES cells (TAG1) [17] whereby Smad2/3 signaling levels are maintained high over time by a tetracycline inducible receptor which is constitutively-active (Alk4*), and repressed by the specific inhibitor SB-431542 (SB) [54]. Activation of p-Smad2/3 signaling directly activates a negative feedback loop (auto shut-off) that includes the direct and immediate activation of extracellular antagonists and intracellular negative regulators [55]. This results in a rapid reduction of the p-Smad2/3 signaling responses at initial stages of stimulation, and it takes time for signaling to achieve a balance between positive and negative regulation and stabilize at intermediate levels. We therefore used the inducible Alk4* system to stimulate signaling but also bypass the extracellular negative feedback, prolonging the initial high signaling activation for longer. Using this system we generated a database of p-Smad2/3 both up- and down-regulated miRNAs under the following three conditions: 1) normal culture with serum (representing conditions known to exhibit physiologic activation of p-Smad2/3 signaling), 2) Smad2/3 activation (via tet-induction of Alk4*) and 3) Smad2/3 repression (using the SB inhibitor). Validation of specific miRNAs by quantitative PCR (qPCR) and further analysis of TGF-β upregulated miRNAs provided evidence of direct p-Smad2/3 regulation in mouse ES cells and early embryos. Promoter/enhancer analysis demonstrated that the largest cluster, pri-miR-341∼3072, is co-regulated by the Smad2/3 transcription partner FoxH1.

## Results

### The Identification of p-Smad2/3-responsive miRNAs

Normally, ES cells secrete several ligands including TGF-β and exhibit activation of Smad2/3 (endogenous signaling). To manipulate p-Smad2/3 levels, we utilized a previously described Alk4*-inducible ES cell line (TAG1) [17] in a time-course experiment. Treatment of this cell line with doxycycline (Dox) induces the expression of a constitutively active Alk4* receptor, resulting in a rapid activation (phosphorylation) of Smad2/3; whereas treatment with the specific inhibitor SB-431542 (termed SB hereafter) blocks the phosphorylation of Smad2/3 and inactivates the pathway [54].

**Figure 1:**
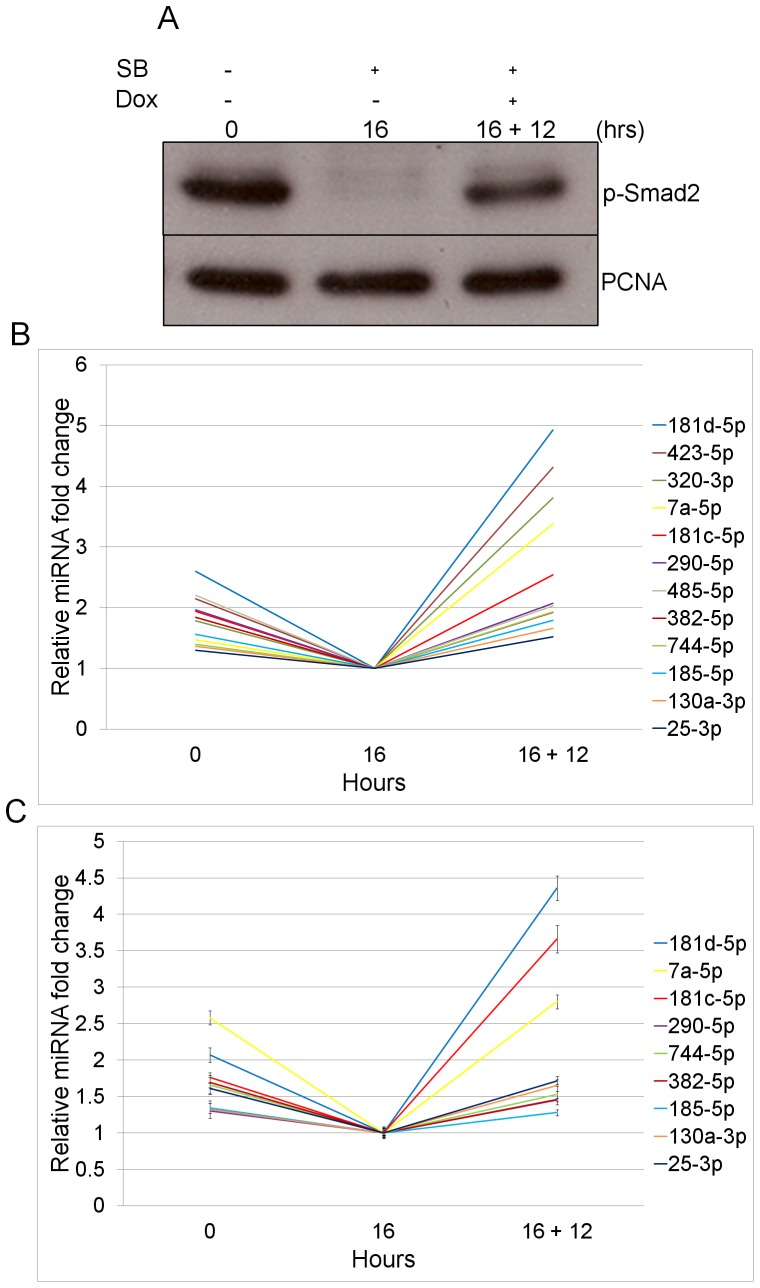
Identification of p-Smad2/3 inducible miRNAs. (**A**) Western blot analysis of p-Smad2 and PCNA (loading control) levels in TAG1 ES cells untreated (0), treated with SB-431542 (SB) for 16 hrs (16) or with SB for 16 hrs followed by doxycycline (Dox) for 12 hrs (16+12). (**B**) Relative fold induction of mature miRNAs in TAG1 cells treated as in (**A**) detected by small RNA deep-sequencing. (**C**) qPCR validation of the deep-sequencing data, normalized to the non-Smad2/3 responsive miR-26a. Error bars represent ±SEM of triplicate reactions.

TAG1 cells were treated under serum free medium for 16 hours (16 hrs SB) with SB to eliminate Smad2/3 activation and thus switch-off endogenous signaling, or treated first with SB for 16 hrs followed by treatment with Dox for 12 hrs (16 hrs SB +12 hrs Dox) to induce Smad2/3 activation. To determine whether Smad2/3 activation under Alk4* induction was in agreement with a more physiological activation, we sequenced miRNAs from ES cells grown in high serum (20%; 0 hr) known to contain TGF-β ligands. This sample contains high levels of p-Smad2 (Figure 1A) and therefore can be used to verify whether the same miRNAs can reproducibly be upregulated by different p-Smad2/3 activation conditions.

As the processing of some pri-miRNA transcripts can be relatively inefficient [56], the induction was performed over a period of 12 hours to allow mature miRNAs to accumulate at significant enough levels to expose clear differences between p-Smad2/3 activation and repression conditions (16 hrs SB treatment). Furthermore, the 12 hour activation includes the period (1–2 hrs) that Alk4* requires to be transcribed and translated after the induction with Dox, and before Smad2/3 are activated and capable of regulating miRNA targets. The long period of inhibition was performed preceding the induction to allow us to see *de novo* responses downstream of p-Smad2/3 activation. Total RNA and protein were subsequently extracted from cells at each time point. Western blot analysis was performed to examine p-Smad2 levels following treatment and to confirm their suppression with SB, their increase with Dox and the steady state levels in the untreated control cells (Figure 1A). To identify the mature miRNAs responsive to these changes in p-Smad2/3 levels, we performed short RNA deep-sequencing using the Illumina platform.

The total number of reads obtained over two runs of deep-sequencing for each sample were 11,930,468 for the 0 hr sample (untreated), 12,356,171 for the 16 hr SB sample and 11,267,363 for the 16 hr SB +12 hr Dox sample. The sequenced reads were aligned to miRBase (release 18) (described in the material and methods) allowing only perfect matches. Under these conditions, the number of reads mapping to mature miRNAs were 3,101,837 for the 0 hr sample, 2,738,932 for the 16 hr sample and 3,302,409 for the 16 hr SB +12 hr Dox sample. Overall, we were able to detect 693 mature miRNAs in the TAG1 ES cells (Table S1). miRNAs showing ≥1.25-fold change in abundance between p-Smad2/3 activation (16 hrs SB +12 hrs Dox) and suppression (16 hrs SB), and also ≥1.25-fold change between p-Smad2/3 steady state levels (0 hr) and suppression (16 hrs SB), were considered as p-Smad2/3 responsive.

After applying Z-test and fold-change analysis (described in the materials and methods), we identified 145 miRNAs showing a significant down-regulation after SB treatment (P≤0.05) and a significant up-regulation after Dox treatment (P≤0.05) (Table S1). Among these miRNAs that followed the p-Smad2/3 activation profile, we selected the most abundant in TAG1 ES cells based on the sum of normalized reads between the pair of samples compared in the previous two criteria (16 hrs SB and 0 hr; 16 hrs SB +12 hrs Dox and 16 hrs SB). This narrowed our selection to 28 miRNAs (Table 1); these miRNAs displayed the lowest Z-score values for 16 hrs SB versus 0 hr test (below −22.0) and the highest Z-score values for 16 hrs SB +12 hrs Dox versus 16 hrs SB test (above 17) (Table S1).

**Table 1:** Identification of Smad2/3 inducible miRNAs by small RNA deep-sequencing and real-time qPCR.

miRNA	TAG1										J1
	Total reads			Normalized reads			Fold-change				
	0 h	16 h	16+12 h	0 h	16 h	16+12 h	16h v 0h		16+12h v 16h		
							DS	qPCR	DS	qPCR	
485-3p	15123	3204	14883	1380	282	1504	−4.89		5.33		
181d-5p	107042	42731	183584	9770	3763	18552	−2.60	−2.07	4.93	4.36	1.09
423-5p	62261	30061	112927	5683	2647	11412	−2.15	1.12	4.31	1.11	1.32
25-5p	15513	8015	29645	1416	706	2996	−2.01		4.24		
320-3p	90928	52727	174984	8300	4644	17683	−1.79	1.04	3.81	1.16	1.2
7a-5p	12989	9111	28023	1186	802	2832	−1.48	−2.58	3.53	2.8	1.07
128-3p	10758	6212	14348	982	547	1450	−1.79		2.65		
181c-5p	11420	6098	13507	1042	537	1365	−1.94	−1.76	2.54	3.66	−1.01
295-5p	15726	10758	22723	1435	947	2296	−1.52		2.42		
543-3p	77103	24436	50833	7038	2152	5137	−3.27		2.39		
298-5p	20568	13599	27069	1877	1198	2735	−1.57		2.28		
323-3p	12677	6410	12510	1157	565	1264	−2.05		2.24		
290-5p	390434	205450	370150	35638	18094	37406	−1.97	−1.3	2.07	1.46	−1.04
485-5p	11901	5602	9914	1086	493	1002	−2.20	−1.42	2.03	1.1	1.09
433-3p	16712	6807	11648	1525	599	1177	−2.54		1.96		
744-5p	16396	12174	20521	1497	1072	2074	−1.40	−1.54	1.93	1.59	−1.09
382-5p	21060	11862	19882	1922	1045	2009	−1.84	−1.69	1.92	1.45	1
672-5p	43113	19036	31043	3935	1676	3137	−2.35		1.87		
669c-5p	15701	12393	19642	1433	1091	1985	−1.31		1.82		
185-5p	26062	17301	27039	2379	1524	2732	−1.56	−1.34	1.79	1.28	1.09
541-5p	28812	19793	30572	2630	1743	3089	−1.51		1.77		
92a-3p	58724	36877	55151	5360	3248	5573	−1.65		1.72		
130a-3p	15364	11642	16887	1402	1025	1707	−1.37	−1.32	1.66	1.65	1.02
363-5p	24461	13906	19919	2233	1225	2013	−1.82		1.64		
291a-5p	28030	21958	29674	2558	1934	2999	−1.32		1.55		
25-3p	140851	111946	148185	12856	9859	14975	−1.30	−1.61	1.52	1.71	1.05
341-3p	14130	9000	11191	1290	793	1131	−1.63		1.43		
382-3p	12866	7865	9310	1174	693	941	−1.70		1.36		

DS  =  Deep-sequencing.

To confirm whether the deep-sequencing results were in agreement with other methods of detection, we selected 12 of these miRNAs (based on assay availability) and performed real-time RT-qPCR from the same total RNA samples that had been sequenced. Figure 1B shows the relative fold-change of these 12 miRNAs according to the deep-sequencing data. Nine of the 12 miRNAs displayed fold abundance changes of ≥1.25 in the qPCR (Figure 1C and Table 1), suggesting that these miRNAs respond to p-Smad2/3. To exclude the possibility that these miRNAs are induced by Dox and not by p-Smad2/3, the same treatments were performed in the TAG1 parental cell line, J1, which lacks the Dox-inducible Alk4* receptor. Only one miRNA, miR-423-5p, was found to respond to the Dox treatment, as its fold abundance in the control J1 cells was greater than 1.25 in the qPCR (Table 1). In summary, by deep-sequencing, we identified 28 p-Smad2/3 responsive miRNAs with a high abundance and in validation by qPCR we verified 9 out of 12 of these miRNAs, indicating that these were valid targets of the TGF-β signaling pathway.

### Identification of miRNAs Directly Induced by p-Smad2/3

To determine whether any of the nine miRNAs are direct targets of p-Smad2/3, we analyzed the levels of their primary transcripts in the presence of the protein synthesis inhibitor cycloheximide (CHX). Under these conditions, an increase in pri-miRNA levels following p-Smad2/3 induction indicates that a miRNA is directly activated and does not depend on the translation of proteins and intermediate transcription factors activated downstream of p-Smad2/3.

**Figure 2:**
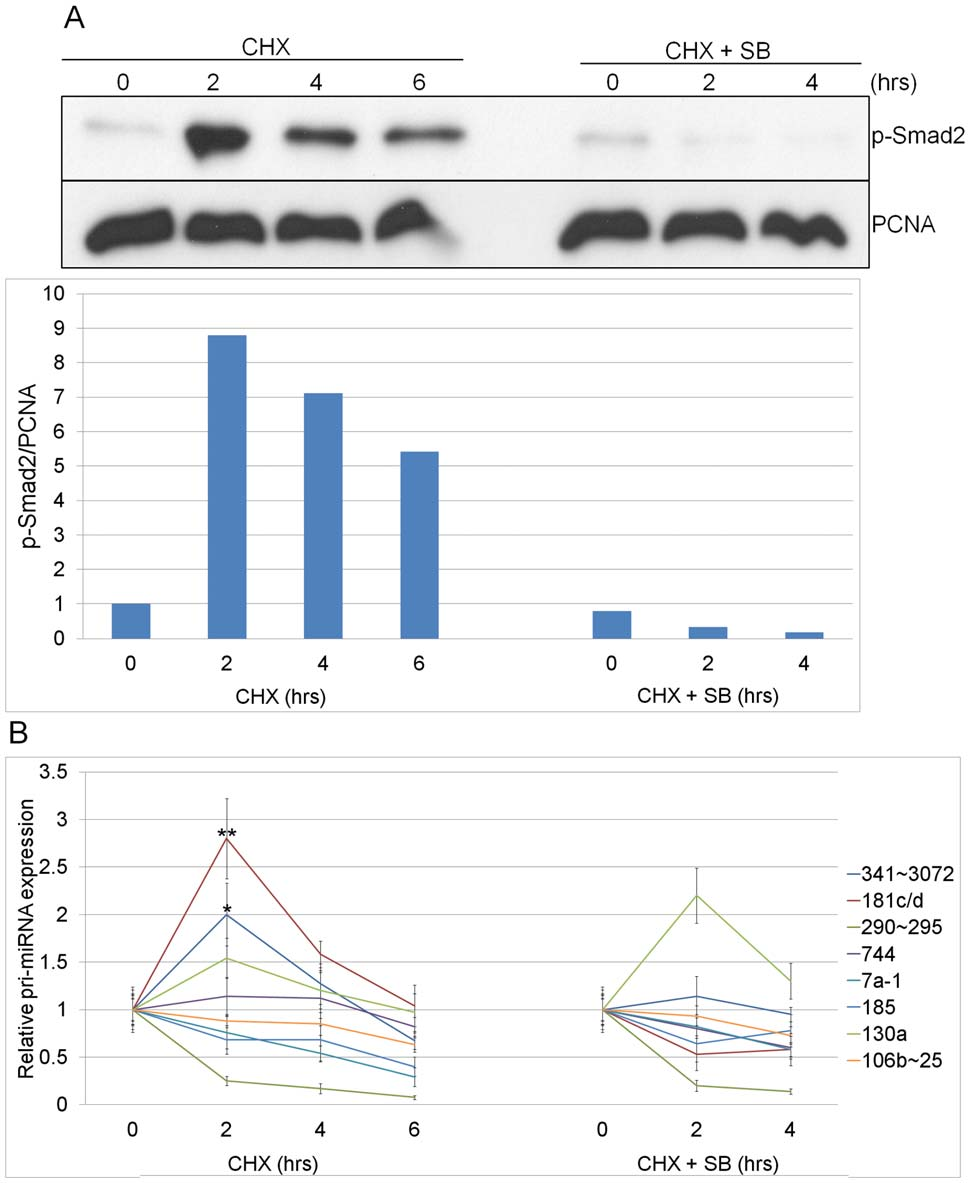
Identification of miRNAs induced directly by p-Smad2/3. (**A**) Western blot analysis of p-Smad2 and PCNA (loading control) levels in TAG1 ES cells pre-treated with SB-431542+ doxycycline (SB+Dox) for 16 hrs, followed by cycloheximide (CHX) treatment for 2, 4, and 6 hrs or CHX+SB for 2 and 4 hrs. The graph presents relative p-Smad2 levels normalized to corresponding PCNA levels. (**B**) qPCR analysis of pri-miRNA expression in CHX (left side) and CHX+SB (right side) treated TAG1 cells. Asterisks denote statistical comparisons of 2 vs. 0 hr time points (Student’s t-test, **p<0.01, *p<0.05). Pri-miRNA levels were normalized to *Gapdh*. Pri-miR-7a-2 was not detected. Average of 3 biological replicates (n = 3) performed in triplicates. Error bars show ±SEM.

TAG1 cells were pre-treated for 16 hrs with Dox to induce and accumulate high levels of Alk4* receptor, in the simultaneous presence of SB to prevent phosphorylation of Smad2/3 by the receptors. This 16 hrs SB+Dox is time point zero (0 hr). Subsequently, the SB was removed and CHX was added to inhibit protein synthesis (CHX without SB) over 2, 4 and 6 hr time points. To assess the effect of CHX alone, after the pre-treatment, we treated cells with CHX in the presence of SB over the same time points (CHX+SB). Total RNA and protein were extracted from cells at each time point and analyzed by western blot (Figure 2A) and qPCR (Figure 2B). P-Smad2 levels were almost undetectable after pre-treatment (0 hr) while at 2 hrs with CHX and without SB, a strong induction (8.8-fold above those at time 0 hr) was observed (Figure 2A). P-Smad2 levels gradually decreased after 4 and 6 hrs, most likely due to a decrease in Alk4* receptor protein levels. In contrast, treatment with CHX+SB did not significantly alter the levels of p-Smad2 over the same time points (Figure 2A).

We performed qPCRs to determine the expression levels of pri-miRNAs at each of the above time points. Table 2 lists the mature miRNAs found to respond to p-Smad2/3 in the deep-sequencing and qPCR validation experiments, and the 9 different pri-miRNA transcripts encoding them. Pri-miRs-341∼3072 and −181c/d showed a significant upregulation (2- and 2.8-fold, respectively) after only 2 hrs with CHX treatment without SB (Figure 2B). Furthermore, levels of both pri-miRNAs decreased after 4 and 6 hrs, in correlation with the decrease in p-Smad2 levels at these time points (Figure 2A and 2B). In the control CHX+SB experiment, neither pri-miR-341∼3072 nor -181c/d changed significantly in expression levels (Figure 2B). The remaining pri-miRNAs did not increase in response to p-Smad2/3 induction, with one pri-miRNA (pri-miR-130a) responding to CHX (Figure 2B). Together, these findings suggest that pri-miRs-341∼3072 and -181c/d are direct targets of p-Smad2/3 transcription.

**Table 2:** Pri-miRNA genes encoding the p-Smad2/3-inducible miRNAs.

miRNA	Pri-miRNA	Host gene (intronic)/intergenic
7a-5p-1	Monocistronic	Hnrnpk
7a-5p-2	Monocistronic	Intergenic
130a-3p	Monocistronic	Intergenic
25-3p	106b∼25	MCM7
†181c-5p	181c/d	Intergenic
†181d-5p	181c/d	Intergenic
185-5p	Monocistronic	RP23-47O21.5
290-5p	290∼295	Intergenic
†382-5p	341∼3072	Intergenic
744-5p	Monocistronic	Map2k4

† Directly regulated p-Smad2/3-responsive miRNAs.

### Characterization of the p-Smad2/3 Inducible pri-miRNA Genes

**Figure 3:**
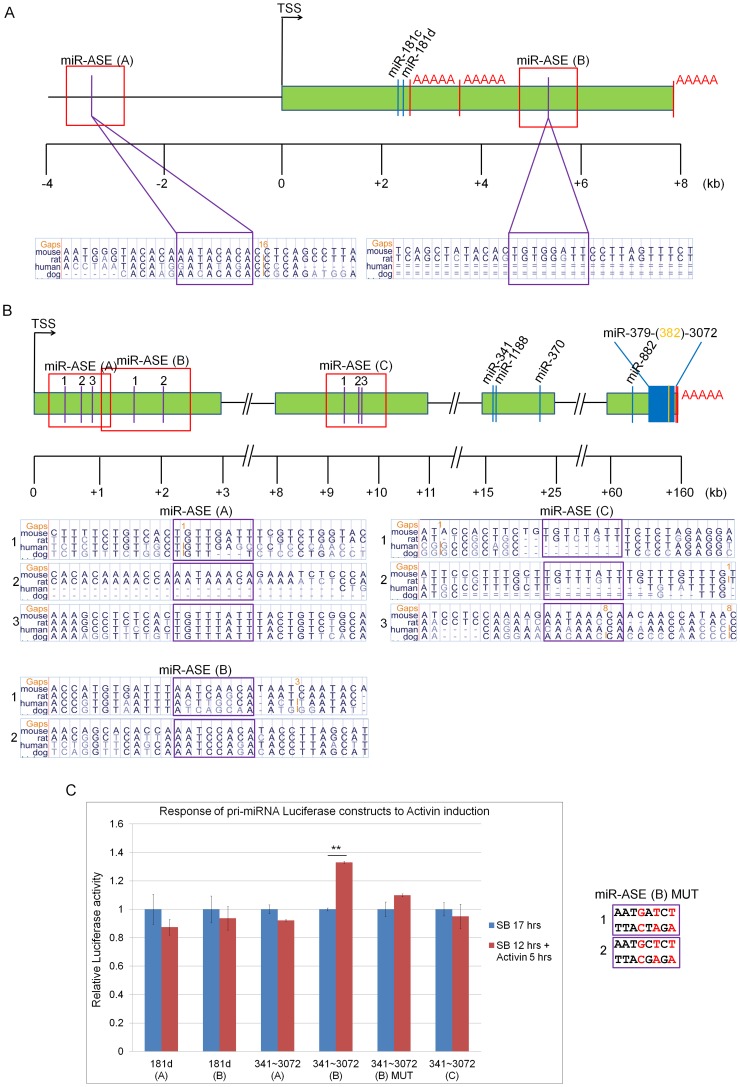
Predicted gene structure of pri-miRs-181c/d and -341∼3072 and validation of FoxH1 binding sites. (**A, B**) Schematic representations of the predicted gene structures of pri-miR-181c/d (**A**) and pri-miR-341∼3072 (**B**). The putative FoxH1 binding sites (Asymmetric Enhancer Elements, ASE) are indicated in purple. Sequence and species conservation of the ASE, in purple boxes, are shown below the respective gene maps. Positions of miRNAs are indicated with blue lines and in yellow for miR-382 (**B**). ‘AAAA’ in red denotes the predicted poly(A) tails. A red box highlights sequence fragments used for each pri-miRNA expression luciferase construct. TSS – transcriptional start site. (**C**) Candidate putative miR-ASE sites were tested by luciferase assay in ES cells treated with SB-431542 (SB) for 12 hrs followed by Activin for 5 hrs or with a further SB treatment of 5 hrs (SB 17 hrs). The nucleotides mutated to generate the miR-ASE(B)MUT construct are presented in the panel to the right of the graph. Student’s t-test was used for the statistical analysis (**p<0.01). Assays were performed in 3 biological replicates measured in quadruplicate (n = 3). Error bars show ±SEM.

To gain further evidence for the direct regulation of pri-miRs-341∼3072 and -181c/d by p-Smad2/3, we investigated the promoters of these primary transcripts. At present, there is little experimental evidence available on the structure of intergenic pri-miRNA genes. One multiplexed approach has been to combine data on the presence of chromatin signatures, CpG islands, ESTs and species comparisons, to predict transcriptional start sites (TSS) to a high degree of probability [28]. Using these data, we made predictions for the gene structures of pri-miRs-181c/d and -341∼3072 (Figure 3A and 3B, respectively).

FoxH1 is a critical co-regulator of p-Smad2/3-dependent target gene activation, and is expressed at high levels throughout early mouse embryogenesis and ES cells [13], [15]. To investigate whether there are putative FoxH1 binding sites (here termed Asymmetric Enhancer Elements, ASE [57]) present in the predicted regulatory regions of pri-miRs-181c/d and -341∼3072, we used the Fuzznuc algorithm to search for the presence of ASE 10 kb upstream and downstream of their predicted TSS. Within this region, we found two ASE for pri-miR-181c/d and eight for pri-miR-341∼3072. The relative position of these ASE, and their species conservation, is presented in Figure 3A and 3B. We also found an abundance of putative Smad Binding Elements (SBE) in close vicinity of these ASE, supporting a Smad-dependent regulation from these ASE. Therefore, we hypothesized that these pri-miRNAs are regulated in a FoxH1-dependent manner. To test this, we analyzed the functionality of these putative ASE sites by generating luciferase reporter constructs in which the enhancer region upstream of the SV40 promoter in the pGL3 mammalian expression vector was replaced with 1–1.5 kb fragments containing single or multiple ASE of pri-miRs-341∼3072 and -181c/d, and tested each group in the luciferase assay (Figure 3C).

Each miR-ASE luciferase construct was transfected into ES cells and treated with SB for 12 hrs to inhibit endogenous signaling. Cells were subsequently treated with Activin for 5 hrs to activate the pathway or with SB as negative control for an additional 5 hrs. We observed a significant upregulation in luciferase activity (1.33-fold; P<0.01) following Activin treatment for miR-ASE(B) of pri-miR-341∼3072 (Figure 3C), indicating that this ASE cluster may be important in the transcriptional regulation of pri-miR-341∼3072. To validate this responsiveness, both ASE sites within this region were mutated (miR-ASE(B)MUT) and tested in luciferase assays. The miR-ASE(B)MUT construct did not elicit any response to Activin treatment (Figure 3C), confirming that this site is a functional ASE. These results suggest that pri-miR-341∼3072 is a direct target of p-Smad2/3 co-regulated by FoxH1.

The putative pri-miR-341∼3072 gene encodes 83 mature miRNAs, of which 8 were among the group of 28 miRNAs with a significant p-Smad2/3 activation profile and high level of expression (Table S2). Moreover, another 26 miRNAs derived from pri-miR-341∼3072 also showed a significant p-Smad2/3 activation profile by deep-sequencing (Table S2). Interestingly, no expression was detected for 11 of the 83 miRNAs in the cluster, suggesting that it is regulated post-transcriptionally, i.e. these miRNAs are inefficiently processed or display a low stability in ES cells.

### The p-Smad2/3-responsive miRNAs are Expressed in Early Mouse Embryos

**Figure 4:**
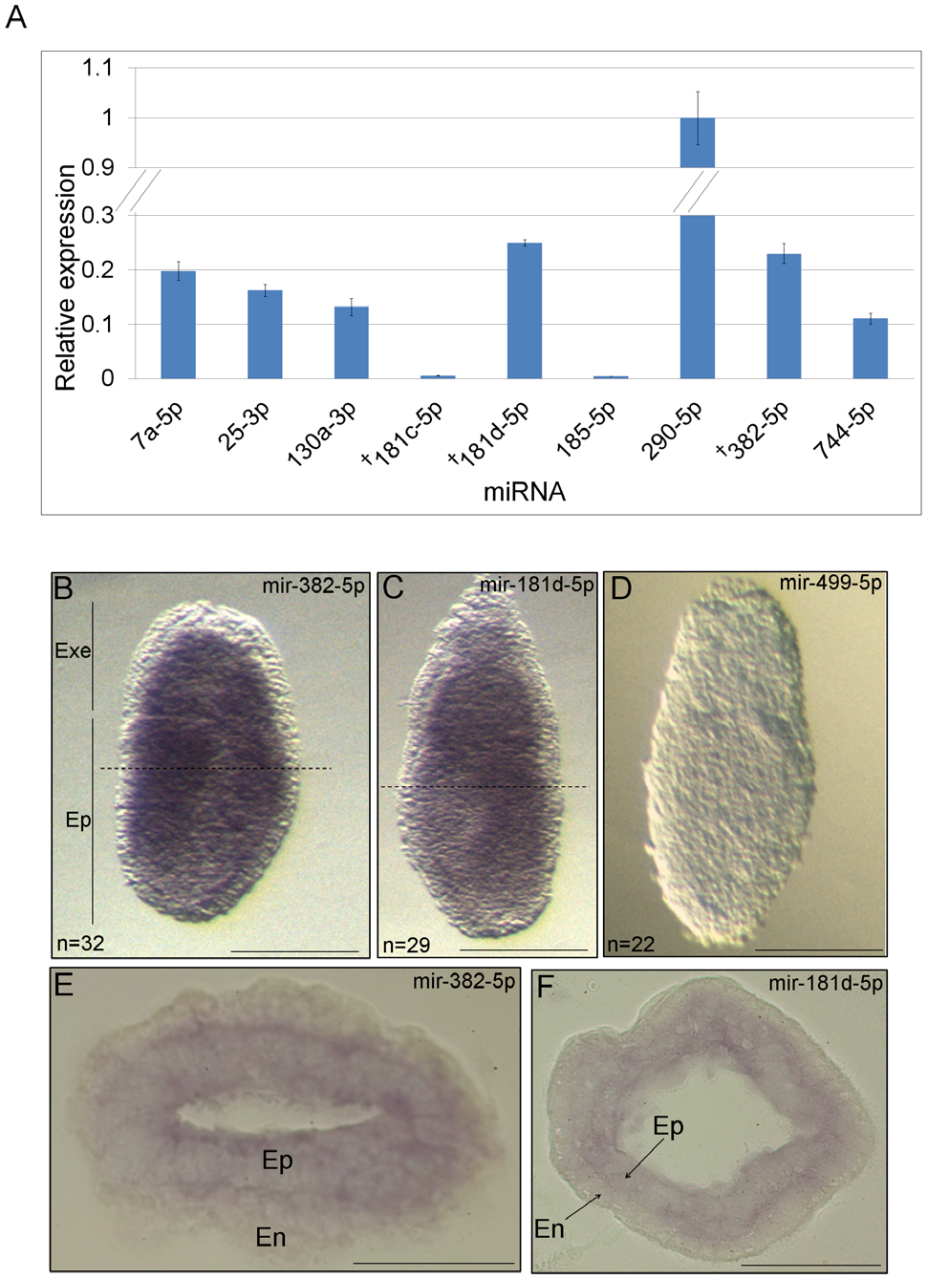
Expression of p-Smad2/3 inducible miRNAs in the early embryo. (**A**) qPCR was performed on RNA from pooled mouse E6.5 embryos to detect the relative expression levels of the p-Smad2/3 inducible miRNAs. Error bars show ±SEM of triplicate assays. ‘^†^’ denotes the direct p-Smad2/3 target miRNAs. (**B–F**) *In situ* hybridizations were performed on E6.5 embryos using Locked Nucleic Acid modified oligonucleotides against miRs-382-5p (**B, E**), -181d-5p (**C, F**) and -499-5p (negative control) (**D**). Images are representative of 32 embryos for -382-5p, 29 for -181d-5p, and 22 for -499-5p as indicated in the images (n). Dotted lines in (**B, C**) indicate positions of sections presented in (**E, F**), respectively. Scale bars for whole mounts indicate 100 µm (**B, C, D**), and for sections 50 µm (**E, F**). Extra-embryonic (Exe), epiblast (Ep) and endoderm (En).

As TGF-β/Nodal signaling plays an essential role in the development of the early mouse embryo, we addressed the *in vivo* relevance of the miRNA targets by first examining the expression of p-Smad2/3-induced miRNAs at embryonic day 6 (E6.5). At this stage Nodal is expressed highly and its function is essential. Total RNA was extracted from these embryos and mature miRNA levels were analyzed by qPCR (Figure 4A). All p-Smad2/3-responsive miRNAs were expressed. Interestingly, miRs-181c-5p and -181d-5p, which are derived from a common primary transcript, showed a considerable difference in expression levels (miR-181d-5p 40-fold higher than -181c-5p), suggesting that differential post-transcriptional processing or stability regulates the levels of one miRNA over the other.

We next investigated the expression pattern of the direct p-Smad2/3 miRNA targets, miRs-181d-5p and -382-5p. For this we performed Locked Nucleic Acid oligonucleotide *in situ* hybridization (ISH) on E6.5 embryos. The ISH demonstrated ubiquitous expression of both miRNAs throughout the epiblast and extraembryonic regions of the embryo (Figure 4B, 4C, 4E and 4F), while no signal was detected for the negative control miR-499-5p (Figure 4D), which is not expressed at this stage of development, confirming the specificity of staining. Nodal is expressed throughout the epiblast and the visceral endoderm in E6 embryos and retracts to the posterior epiblast during gastrulation at E7.5 of development [55]. Broad initial activation of miRNAs by Nodal and relatively high miRNA stability is consistent with the wide expression of these miRNAs in E6.5 stage embryos.

**Figure 5:**
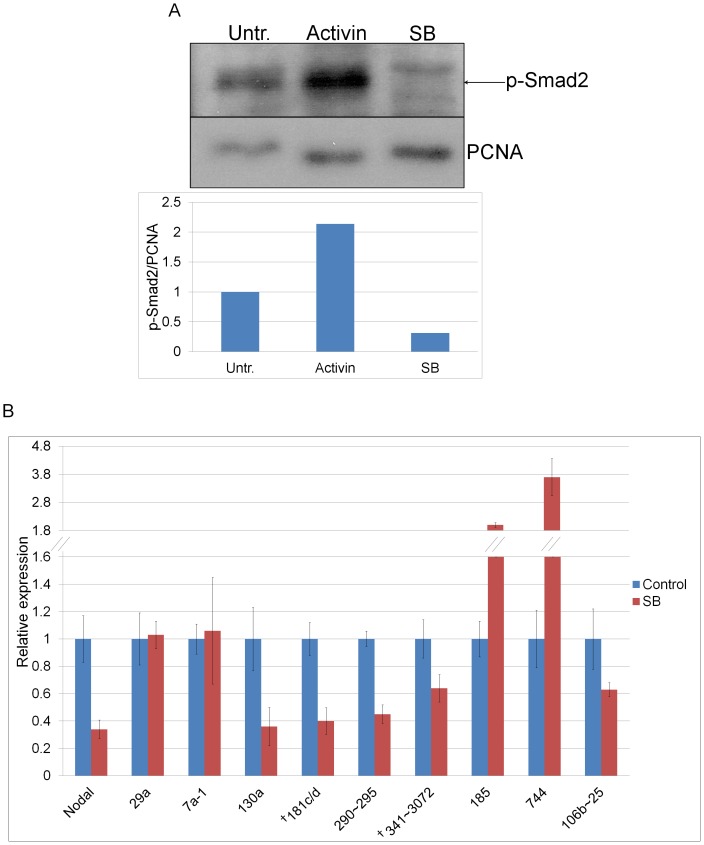
p-Smad2/3 inducible miRNAs respond to changes in p-Smad2/3 signaling in the early embryo. (**A**) Western blot analysis of p-Smad2 and PCNA (loading control) levels in pooled E6.5 embryos untreated (Untr.) or treated with Activin or SB-431542 (SB) for 3.5 hrs. The graph presents p-Smad2 levels normalized to corresponding PCNA levels. (**B**) qPCR analysis of p-Smad2/3 inducible pri-miRNAs on RNA isolated from pooled E6.5 embryos treated with SB or 0.1% DMSO (SB solvent, Control) for 5 hrs. The relative expression was calculated by normalizing to *Gapdh* for Nodal and to pri-miR-17 (non-Smad2/3 responsive control) for the pri-miRNAs. Pri-miR-7a-2 was not detected. Error bars show ±SEM of triplicate assays. ^†^Denotes direct p-Smad2/3 target miRNAs.

To determine whether the p-Smad2/3 induced miRNAs expressed in the early embryo (Figure 4A) were also responsive to p-Smad2/3 levels, E6.5 embryos were treated with SB or control DMSO (SB solvent) and total RNA and protein were extracted. Treatment with 20 µM SB for 3.5 hrs resulted in a 2-fold reduction in p-Smad2 protein levels compared to control DMSO-treated embryos (Figure 5A). After confirming that p-Smad2 levels in the embryo were responsive to SB treatment, we performed qPCR analysis with the RNA extracted from embryos treated for 5 hrs with SB and DMSO (Figure 5B). The Nodal gene, which is a direct target of its own signaling and p-Smad2/3, was downregulated by 3-fold in response to SB, confirming that this treatment was effective. On the contrary, pri-miR-29a, which according to the deep-sequencing data is non-responsive to p-Smad2/3 activation, remained unchanged, confirming that the response was specific to p-Smad2/3 regulated pri-miRNA. Both pri-miRs-341∼3072 and -181c/d were downregulated with SB indicating that, like in ES cells, they are also upregulated by p-Smad2/3 in the early mouse embryo (Figure 5B). Interestingly, pri-miRs-185 and -744 showed increased expression with SB treatment suggesting that they are repressed by Nodal in the embryo. Collectively, our results demonstrate that the majority of pri-miRNAs induced in ES cells are expressed and upregulated by p-Smad2/3 also in the early mouse embryo.

### Identification of Putative Target Genes of the p-Smad2/3-induced miRNAs in ES Cells

**Figure 6:**
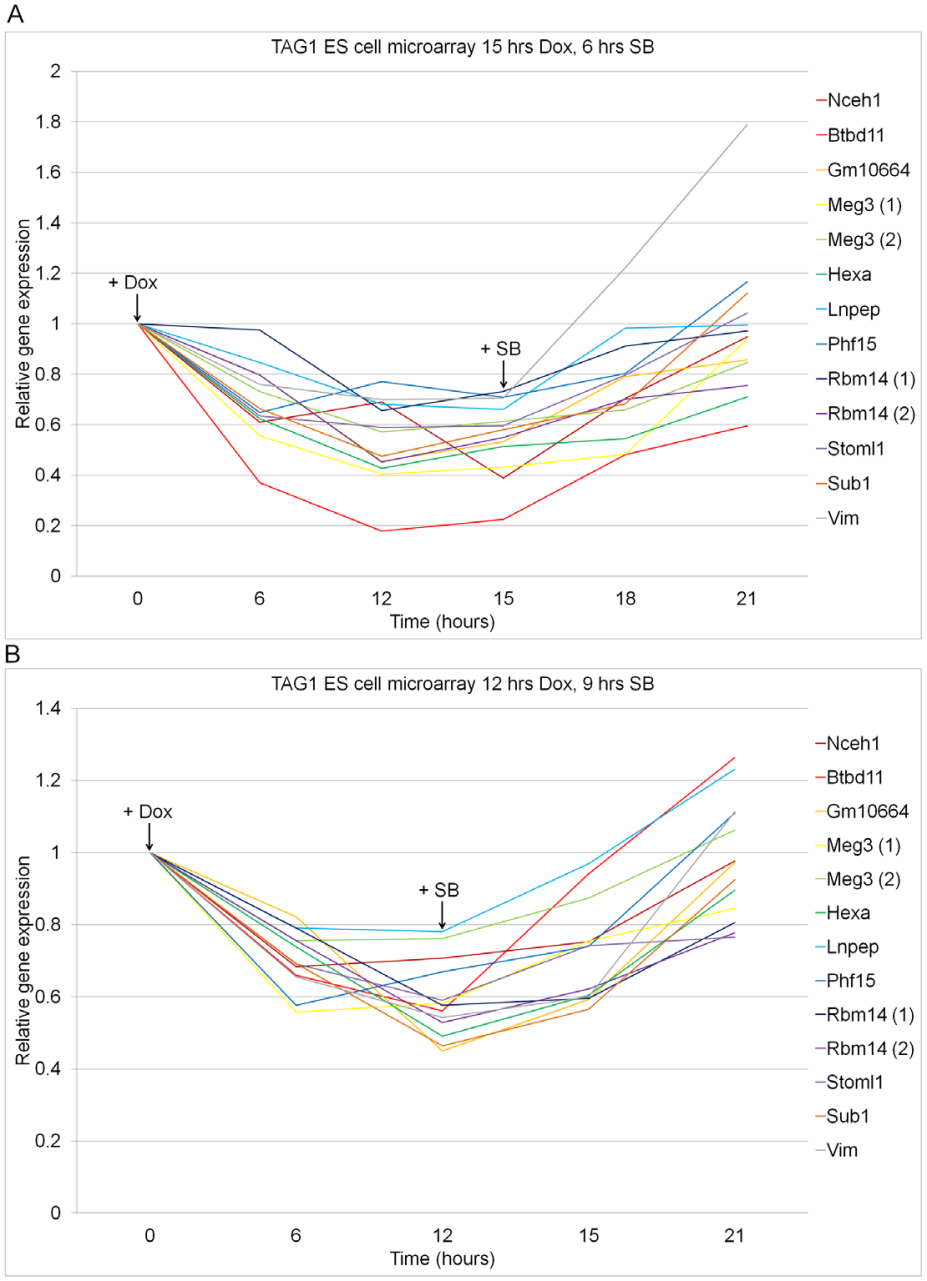
Expression profile of negatively regulated p-Smad2/3 target genes in TAG1 ES cells. Analysis of Affymetrix expression microarray on RNA extracted from TAG1 ES cells either pre-treated for 6 hrs with SB-431542 (SB) followed by 15 hrs with doxycycline (Dox), and 6 hrs with SB (**A**) or pre-treated for 6 hrs with SB followed by 12 hrs with Dox, and 9 hrs with SB (**B**). Genes with >1.2-fold decrease in expression after 15 hrs Dox treatment (15 hrs vs. 0 hr) and >1.2-fold increase after 6 hrs SB treatment (21 hrs vs. 15 hrs) (**A**) and after 12 hrs Dox (12 hrs vs. 0 hr) and 9 hrs SB treatment (21 hrs vs. 12 hrs) (**B**) are presented in the graph. All time points were normalized to the 0 hr non-induced control. Several genes were measured by multiple probe sets as indicated.xl.

To investigate the hypothesis that p-Smad2/3 activate miRNAs that repress downstream gene expression, Affymetrix microarray data on p-Smad2/3 downregulated genes in the TAG1 ES cells was re-analyzed (ArrayExpress, accession E-TABM-574) [17]. In these experiments TGF-β signaling was manipulated over similar time periods with SB and Dox in two independent time-course experiments. In the first analysis, cells were pre-treated with SB overnight (0 hr) to switch-off signaling, treated with Dox for 15 hrs to reach peak p-Smad2/3 activation (time point 15 hrs) and subsequently with SB for 6 hrs to repress p-Smad2/3 (time point 21 hrs) (Figure 6A). Genes showing a reduction in their expression (>1.2-fold) with Dox at 15 hrs compared to 0 hr, and genes showing an increase (>1.2-fold) after SB treatment (21 hrs vs. 15 hrs) were considered as target genes repressed by p-Smad2/3 (Figure 6A). We also analyzed gene expression levels in a second time-course experiment to test the reproducibility of these findings (Figure 6B). In this experiment, cells were also pre-treated with SB overnight (0 hr), but followed by a 12 hr Dox treatment and subsequently SB for 9 hrs. A 1.2-fold cut-off was also used to determine whether the same genes showed a reduction in expression after 12 hrs (12 hrs vs. 0 hr) and an induction after 9 hr SB treatment (21 hrs vs. 12 hrs) (Figure 6B). The 11 genes we identified followed the same trend and showed a >1.2-fold change in expression in response to the Dox and SB treatments in both experiments, demonstrating that our findings are reproducible. The expression levels of these genes changed inversely to p-Smad2/3 and to the induced miRNAs, suggesting that they are candidate targets of the induced miRNAs. Information on the 11 identified downregulated target genes is summarized in Table S3. Utilizing two of the most prominent miRNA target prediction algorithms, Diana microT [58] and microCosm [59], [60], we searched *in silico* for the presence of binding sites within the p-Smad2/3 downregulated genes. Six of these contained potential binding sites for at least one of the directly induced p-Smad2/3-responsive miRNAs (Table 3). This analysis supports the hypothesis that the p-Smad2/3 directly induced miRNAs are involved in the downstream repression of genes by this pathway.

**Table 3:** miRNA target predictions for negatively regulated p-Smad2/3 target genes.

Gene symbol	Predicted miRNA target sites (algorithm)	
	microCosm	Diana microT
Btbd11	–	–
Gm10664	382-3p, 377-3p	–
Hexa	–	–
Lnpep	–	–
Meg3	–	–
Nceh1	–	181c-5p
Phf15	–	181d-5p, 181c-5p, 381-3p
Rbm14	–	–
Stoml1	341-3p	–
Sub1	–	323-3p
Vim	382-5p (x2), 376a-3p	–

– No predicted miRNA target sites.

## Discussion

Although miRNAs are emerging as key post-transcriptional modulators of gene expression, and like other genes they exhibit spatial and temporal regulation, little is known about the transcription factors and pathways that control their expression [26], [27], [28]. Here we performed RNA deep-sequencing in mouse ES cells and generated a database of TGF-β/Smad2/3 regulated miRNAs (both up- and down-). We identified members that are directly upregulated by p-Smad2/3 and confirmed their *in vivo* relevance by showing that their expression in the early embryo depends on Smad2/3 activation.

In an earlier study, we developed an inducible (Tet-On) system in mouse ES cells (TAG1) to manipulate the levels of activated Smad2/3, transducers of TGF-β/Activin/Nodal signaling. We used it to perform time-course experiments and expression profiling with microarrays to generate a database of p-Smad2/3 responsive genes over time [17]. This database includes a number of p-Smad2/3 repressed genes. The repressive function of p-Smads on target genes is not well understood and it remained unknown whether this can be mediated by the activation of miRNAs. In this paper, we utilized the same inducible mouse ES cells and RNA deep-sequencing to identify immediate early responsive miRNAs downstream of TGF-β/Smad2/3 signaling. Analysis of pri-miRNA levels in response to p-Smad2/3 induction and in the absence of protein synthesis (cycloheximide treatment), demonstrated that pri-miRs-181c/d and -341∼3072 are direct p-Smad2/3 targets. Furthermore, promoter/enhancer luciferase reporter experiments revealed that pri-miR-341∼3072 is co-regulated by FoxH1, a Smad2/3 transcription factor partner. Bioinformatic analysis revealed the presence of putative binding sites of the above miRNAs within the p-Smad2/3 downregulated genes, supporting the hypothesis that the repression of gene expression downstream of TGF-β signaling is mediated by the direct activation of miRNAs. This is a translation-independent mechanism, and therefore fast acting and efficient, as the Smad effectors can transcribe several miRNAs in clusters that together target the same genes, to repress downstream gene expression.

The major reason for using the TAG1 cell line to identify p-Smad2/3 responsive miRNAs is that p-Smad2/3 stimulation occurs via the tet-induction of the constitutive receptor Alk4*. This bypasses the extracellular negative feedback mechanisms activated directly and immediately by p-Smad2/3 allowing high signaling responses to be maintained over a long period of time. Furthermore, we evaluated and compared the p-Smad2/3 induced miRNAs under Alk4* induction with that of “untreated” TAG1 ES cells cultured in high serum (20%). These latter conditions contain high levels of ligands, including TGF-β, secreted by the ES cells, and exhibit physiological activation of signaling, shown by the presence of high levels of p-Smad2 (Figure 1A). The majority of the p-Smad2/3 responsive miRNAs we identified were present in both conditions and showed higher responses under constitutive Alk4* induction compared to the physiological activation in the untreated cells (Figure 1B and 1C). These data confirm that the constitutive Alk4* activation conditions boost the p-Smad2/3 transcriptional responses over the 12 hour period. Furthermore, the above findings show that the response of miRNAs downstream of p-Smad2/3 is reproducible under physiological and Alk4* induced conditions.

We identified a total of nine miRNAs (miR-181d-5p, -7a-5p, -181c-5p, -290-5p, -744-5p, -382-5p, -185-5p, -130a-3p and -25-3p) that were upregulated in response to p-Smad2/3 induction. miR-181 family members and miR-382 have been previously reported to respond to TGF-β/Activin treatment in different cells and tissues [46], [61], [62], [63]. Specifically, miR-181b was activated in response to TGF-β signaling in hepatocellular carcinoma cells, and siRNA knockdown of Smad4 resulted in a decrease in mature miR-181b levels [63]. In addition, miRs-181a/b are activated in response to TGF-β signaling in breast cancer cells [46]. However, in this study siRNA knockdown of Smad4 actually increases mature levels of miR-181 family members, consistent with a Smad4-independent role for Smad effectors, as demonstrated by previous studies [44], [45], [46]. This suggests distinct commitments of Smad2/3 in either the transcriptional (Smad4-dependent) or post-transcriptional (Smad4-independent) regulation of miR-181 family members.

Interestingly, several studies have demonstrated the deregulation of the miR-181 family [64], [65], [66], [67], [68], [69], [70] and members of the pri-miR-341∼3072 cluster [71], [72], [73], [74], [75], [76], [77], [78], [79], in various forms of cancer. Specifically, miR-181 family members (including miR-181c) were found to be upregulated in hepatocarcinoma stem cells [64] and are misexpressed in different forms of leukemia [68], [69], [70]. Furthermore, for example, miR-382 is overexpressed in multiple myeloma disease [71], while miR-485 is downregulated in ovarian cancer [75]. As deregulation of the TGF-β pathway underlies tumor suppression in normal epithelia tissue and TGF-β activation within tumors promotes epithelial-to-mesenchymal transition (EMT) and metastasis, the identification by our studies that these miRNAs are direct targets of the TGF-β pathway provides a previously unknown link suggesting that they are downstream mediators of this pathway in carcinogenesis and cancer progression. Furthermore, miR-382 has already been shown to be a crucial target of TGF-β in EMT of human renal epithelial cells [62], supporting the hypothesis that the identified direct miRNA targets of TGF-β mediate the pathway’s function.

Our findings that pri-miR-341∼3072 was upregulated after 2 hrs of signal activation in the absence of protein synthesis (Figure 2B), that changes in its transcript levels mirrored changes in p-Smad2 levels (Figure 2A and 2B), along with the promoter/enhancer analysis (luciferase reporter studies) and the identification of Smad2/3 enhancers for pri-miR-341∼3072 (Figure 3C), demonstrate that this gene is directly upregulated by p-Smad2/3 in ES cells. However, in the absence of protein synthesis, six miRNAs (miRs-7a-1, -290-5p, -744, -185, -130a and -25-3p) do not show an increase in their primary transcript levels during Smad2/3 activation, suggesting that miRNA upregulation occurs either via a post-transcriptional mechanism or indirectly via transcription factors activated by p-Smad2/3. It is also possible that we did not identify all of the direct p-Smad2/3 miRNA targets in these experiments. The reason for this is that, in the absence of protein synthesis, the peak of Smad2/3 activation is short (2 hrs; Figure 2A) and the proteins involved in the basic transcription machinery of the cell are unstable and decline fast. Therefore, only rapidly induced and stable pri-miRNA transcripts can be detected during this time. This may have prevented some of the pri-miRNA transcripts to accumulate at detectable levels and become validated as directly induced targets. Nevertheless, pri-miR-341∼3072 is a novel pri-miRNA encoding 83 miRNAs, of which 34 follow the p-Smad2/3 activation profile. These 34 miRNAs represent 23% of the 145 miRNAs that were identified by deep-sequencing to be Smad2/3 responsive, indicating that pri-miR-341∼3072 is a key miRNA target of TGF-β signaling.

FoxH1 is a critical co-factor of p-Smad2/3-dependent target gene activation during early mouse development [80]. Bioinformatic analysis of our two directly regulated pri-miRNA genes predicted the presence of multiple FoxH1-binding sites (ASE) within close proximity of their TSS (Figure 3A and 3B). Promoter/enhancer analysis with luciferase reporter assays, showed that pri-miR-341∼3072 is regulated by an ASE cluster downstream of its predicted transcription start site (Figure 3C) suggesting that this cluster is co-regulated by p-Smad2/3-FoxH1 during early development. The 1.33-fold induction of pri-miR-341∼3072 miR-ASE(B) by Activin treatment in the luciferase experiments (Figure 3C) was modest compared to other reporter constructs that contain multiple copies of regulatory elements (e.g. CAGA-luciferase). However, this induction is physiological and significant as the construct is minimal, i.e. it contains only one pair of ASE consensus sequences and is devoid of the rest of the promoter and additional copies of this enhancer. It is known that ASE sequences function in pairs and the majority of Smad2/3-FoxH1 target genes usually contain multiple ASE [80]. This appears to be true for the pri-miR-341∼3072 ASE (Figure 3B) suggesting that the modest luciferase activation might be due to the fact they were tested in isolation. It is also possible that there are additional ASE in more distal regions that we have not examined. These findings, however, provide further evidence that pri-miR-341∼3072 is directly activated in response to p-Smad2/3 induction in mouse ES cells and during early embryonic development, as FoxH1 is essential in mediating Smad2/3 transcription downstream of Nodal, an essential TGF-β ligand at early stages (e.g. E6.5) of mouse development [13], [14].

We further investigated whether the miRNAs we identified are also induced by p-Smad2/3 in E6.5 mouse embryos, as many of the genetic differentiation pathways that are regulated by Nodal at this stage of development are also activated in ES cells [17], [55]. The expression of miRs-382-5p and -181d-5p in E6.5 embryos, along with the fact that their primary transcripts were downregulated in response to SB inhibitor treatment at this stage, confirmed that both pri-miRNAs are also regulated by the Nodal-Smad2/3 pathway during development. As ES cells derive from the inner cell mass of the blastocyst, it would be interesting in future experiments to investigate the role of Nodal signaling and that of the Nodal induced miRNAs identified here, in pre-implantation development.

During the preparation of this manuscript, Fu and colleagues published a study which identified miRNAs activated after 3- and 5-day treatment of mouse ES cells with Activin and Wnt3a [61]. In these experiments, miR-181c remained upregulated by >2-fold after Activin treatment, suggesting that not only is its induction under the control of p-Smad2/3 but also its maintenance. Furthermore, the over-expression of a pool of six miRNAs, including miR-181c, promoted the differentiation of ES cells into the endoderm lineage suggesting that this miRNA is a candidate involved in the well known function of Nodal to induce endoderm. Future studies would address the role for miR-181d, derived from the same primary transcript as miR-181c, *in vivo.*

In order to identify candidate downstream target genes of the directly p-Smad2/3-induced miRNAs, we utilized microarray data from previous experiments [17] in which TAG1 ES cells were manipulated to induce or inhibit p-Smad2/3 levels. We identified a group of 11 genes, which were repressed upon p-Smad2/3 induction and upregulated in response to p-Smad2/3 repression over equivalent time periods in a reproducible manner in two time-course experiments (Figure 6). We therefore hypothesized that these genes might be repressed by the p-Smad2/3-responsive miRNAs as they show an opposite response to changes in p-Smad2/3 levels to that of the miRNAs. Analysis of these candidate target genes using miRNA target prediction algorithms revealed that 6 out of the 11 genes contained predicted miRNA binding sites, with 3 of these 6 genes containing more than one predicted site. The absence of any predicted target sites in the other genes may be as a result of inaccuracies in the target prediction software, as the rules governing miRNA-target site interaction are still poorly understood, or alternatively, they may be regulated by a miRNA independent mechanism. In addition, this list of candidate miRNA targets is not exhaustive as miRNAs can function by translational inhibition [20], [25]; such miRNA targets may only be revealed by analyzing protein levels. Interestingly, there were 8 predicted binding sites for miRNAs derived from the pri-miR-341∼3072 gene cluster, suggesting that this cluster may play an important role in controlling gene repression downstream of Smad2/3 signaling activation. The majority of the 11 p-Smad2/3 downregulated genes we identified here have not been previously linked with TGF-β/Smad2/3 signaling, with the exception of *Vim,* which is regulated by the TGF-β pathway during the EMT transition of many cell types [6], [7], including pancreatic [81] and gastric [82] cancer. However, here we reveal the possible involvement of miRNAs in the downregulation of immediate early targets by TGF-β/Smad2/3 signaling, providing a novel mechanism employed by the pathway to silence genes in ES cells and possibly in an early embryonic cell context.

Determining the targets of the multiple miRNAs generated from the TGF-β-activated pri-miR-341∼3072 gene by experimental validation of our predictions will harness our understanding of what are the key mediators of TGF-β pathway function in early development. As TGF-β signaling plays a major role throughout development and in adult tissue homeostasis, it is involved in congenital and adult diseases such as cancer. Understanding the role of this pathway in miRNA regulation will shed light on the mediators of its function and malfunction, and open new therapeutic approaches for cancer treatment.

## Materials and Methods

### Ethical Statement

All experiments on animals were performed under a UK Home Office Animal licence and approved by the Imperial College ethical review committee.

### Small RNA Deep-sequencing, Alignment and Data Analysis

For a description of RNA extraction for the deep-sequencing, see below. Libraries for deep-sequencing of miRNAs by Illumina were prepared from 10 µg total RNA according to the small RNA preparation protocol v1.0 from Illumina. The size of the libraries produced was confirmed by electrophoresis in an Agilent 2100 Bioanalyzer using a DNA1000 chip (both from Agilent Technologies, UK). Single-end sequencing reads of 36bp were generated from these libraries in a GAII Illumina Genome Analyzer.

The sequencing reads were trimmed from the adaptor using the FASTQ/A Clipper from the FASTX tool kit (http://hannonlab.cshl.edu/fastx_toolkit/). Only reads with a length greater than 16 bases were selected for mapping. Since the main aim of this work was to measure miRNA expression with high confidence, alignments to miRBase were made by allowing only perfect matches. Bowtie (version 0.12.7) [83] was used to align first the selected reads to the miRNA mature sequences for *Mus musculus* included in the mature.fa file from miRBase (version 18). The default parameters were used except for the following: “–seedmms 0–seedlen 60–maqerr 10 –m 2000000 –k 2000000”. Reads mapping to a unique position were assigned to the particular miRNA. Reads mapping to several positions were assigned to the miRNA with the same length whenever possible or to the miRNA with the closest length. In case that more than one miRNA fulfilled this requirement the read was then assigned to each of these miRNAs.

Reads resulting unmapped to mature forms were subsequently aligned to the miRNA precursor sequences for *Mus musculus* included in the hairpin.fa from miRBase (version 18). The parameters used were the same as described before. In order to assign the aligned reads to mature forms produced from the matching precursor, the coordinates for all mature forms derived from the 5p and the 3p arms of all miRNA precursors were determined. For this mature forms for *Mus musculus* extracted from the mature.fa file from miRBase (version 18) were mapped to the precursor sequences (*Mus musculus* haipin.fa, miRBase, version 18) using Bowtie and the same parameters described previously. These coordinates were then used to compare and assign the reads mapped to precursors to the corresponding mature form derived from these precursors. In order for a read to be assigned to a certain mature form, total or partial overlap to the specified coordinates was required. Under these conditions, reads mapping to one position were assigned to the respective mature miRNA. Since some mature forms can be produced from more than one precursor, reads mapping across all precursors were identified and assigned only once to the corresponding mature miRNA. For any remaining reads mapping to several positions in the hairpin.fa, all positions were accepted.

We only considered a miRNA to be expressed if 10 or more reads were found across all 3 samples. The total reads for each mature miRNA identified was normalized to the total reads for the respective sample and multiplied by 1×10^6^ in order to scale the values to a normal range (reads per million). In order to identify miRNAs responding to changes in p-Smad2/3 levels, we required their expression to be decreased in the 16 hr SB-431542 (SB) treated sample (16 hrs) compared to untreated (0 hr) and increased in 16 hr SB +12 hr doxycycline (Dox) (16+12 hrs) versus 16 hr treated sample. For each comparison we used the raw data to calculate Z-scores as described previously [84], [85] and assess the statistical significance of the differences detected between conditions. Also fold-changes were calculated using the normalized data. We selected miRNAs fulfilling the following criteria: (i) fold-change ≤−1.25 and Z-score ≤−1.96 (for a P≤0.05) in 16 hr compared to 0 hr; (ii) fold-change ≥1.25 and Z-score ≥1.96 (for a P≤0.05) in 16+12 hr treated cells compared to 16 hr treatment. This list of miRNAs was further narrowed to the most abundant by requiring the fulfillment of a third condition as follows: the sum of normalized reads between the pair of samples compared in the previous two criteria (16 hrs and 0 hr; 16+12 hrs and 16 hrs) is ≥1,000 in each case.

The same analysis was repeated using quantile normalization, which was very recently reported to be one of the best methods for miRNA-seq normalization [86]. Results obtained were highly comparable to our first analysis, demonstrating the robustness of the data.

Sequencing data has been submitted to the Gene Expression Omnibus (GEO) (www.ncbi.nlm.nih.gov/geo; accession ID: GSE39994).

### RNA Extractions, RT-PCR and Real-time qPCR

For detection of mature miRNAs by deep-sequencing and real-time qPCR, total RNA was extracted with the miRVana miRNA isolation kit (Life Technologies, USA). Real-time qPCR on mature miRNAs was performed using the Taqman miRNA assay (Life Technologies). Control reactions containing no reverse transcriptase were performed to test for DNA contamination. *Gapdh* or non-Smad2/3 responsive miRNA genes were used as references for all of the qPCR reactions. The non-Smad2/3 responsive control miRNAs were initially identified by deep-sequencing and were subsequently validated by qPCR. All qPCR reactions were performed in triplicate on a BioRad CFX96 Real-time System and data was analyzed in the BioRad CFX Manager software with the comparative C_t_ method.

For real-time qPCR assays to detect pri-miRNAs and mRNAs in ES cells, RNA was extracted using the RNeasy mini kit (Qiagen, Germany) with on-column DNase digestion. cDNA was generated with SuperScriptIII reverse transcriptase (Invitrogen, UK) and the random hexamer primer for all reactions, except for the pri-miRNA experiments in embryos for which gene-specific primers were used (Table S4). For pri-miRNA and mRNA analysis in embryos, total RNA was isolated using the miRVana miRNA isolation kit (Life Technologies, USA) as it retains all RNA fractions, allowing analysis of precursor and mature miRNA species. Real-time qPCR reactions were performed with the Sybr Green Quantitect PCR kit (Qiagen, Germany) with the following PCR cycling parameters: 94°C for 15 mins, 45 cycles of 94°C for 15s, 55°C for 30s and 72°C for 30s. Pri-miRNA primers (Table S4) were designed as described previously [87].

### Cell Culture

All ES cell lines were maintained in DMEM (Invitrogen, UK) containing 15% Foetal Bovine Serum (FBS) (Source Bioscience, UK) and Leukemia Inhibitory Factor (LIF) (homemade), 2mM L-Glutamine, 1% Penicillin-Streptomycin, 0.1nM b-Mercaptoethanol at 37°C in a 5% CO_2_ humidified incubator.

For identification of p-Smad2/3 inducible miRNAs in TAG1 and J1 (gift from Anton Wutz, Cambridge, UK) ES cells, p-Smad2/3 inhibitions and inductions were performed using 30 µM SB-431542 (SB) (Sigma, UK) [54] and 1 µg/µl doxycycline (Dox) (Clontech, UK), respectively. Control cells were maintained in media containing DMEM with 20% FBS and 2 x LIF, and SB and Dox treated cells were cultured in DMEM with 20% Knockout Serum Replacement (KSR) (Gibco, UK) and 2 x LIF. For identification of direct p-Smad2/3 targets, TAG1 ES cells were cultured in DMEM with 20% KSR and 2 x LIF. Cells were pre-treated with 60 µM SB and 1.5 µg/µl Dox for 16 hrs after which media was replaced with DMEM containing 100 µg/µl cycloheximide (CHX) (Calbiochem, UK) for 2, 4 or 6 hrs. Control non-induced cells were treated for 2 or 4 hrs with CHX and 60 µM SB.

### Western Blot

ES cells and E6.5 embryos were lysed with RIPA buffer (150mM NaCl, 50mM Tris [pH8.0], 0.5% Deoxycholate, 0.1% SDS, and 1% NP-40) containing phosphatase (Sigma, UK) and protease (Roche Applied Science, Germany) inhibitor cocktails. 30 µg protein was loaded onto each lane of the SDS-PAGE gels. For ES cell lysates, the following primary antibodies were used: rabbit p-Smad2 (1∶500) (Cell Signaling Technology, USA) and mouse PCNA (1∶5,000) (Santa Cruz Biotechnology, USA). Secondary antibodies used: HRP-conjugated anti-rabbit (1∶1,000) and anti-mouse (1∶3,000) (GE Healthcare, UK). For embryo lysates, primary antibodies used: rabbit p-Smad2 (1∶500) (Cell Signaling Technology, USA) and PCNA (1∶10,000) (Millipore, USA). Secondary antibodies used: HRP-conjugated anti-mouse (1∶10,000) (GE Healthcare, UK) and HRP conjugated anti-rabbit (1∶5,000) (Calbiochem, UK). Quantitation of protein bands was performed on scans of films using ImageJ (http://rsbweb.nih.gov/ij/).

### Embryo Culture

For inhibition of p-Smad2/3 signaling, E6.5 embryos from CD1 inter-cross litters were dissected in ice-cold PBS supplemented with 1% KSR (Gibco, UK) and incubated in NDiff N2B27 (Stem Cells Inc., USA) containing 20 µM SB-431542 (SB) (Sigma, UK), 10ng/mL Activin or 0.1% DMSO at 37°C and 5% CO_2_ in a humidified incubator. For western blot analysis, embryos were treated with Activin A (Sigma, UK) or SB for 3.5 hrs. For qPCR analysis, embryos were treated with SB for 5 hrs. Nearly 100 embryos from each treatment were pooled prior to RNA or protein extraction. For analysis of mature miRNA expression in untreated E6.5 embryos, RNA was extracted from nearly 100 embryos of the CD1 inter-cross litters and pooled before the cDNA synthesis.

### In Situ Hybridizations and Cryosectioning

Whole mount in situ hybridizations (WISH) were performed on E6.5 embryos from Hex-GFP mice litters. These mice were used in order for the orientation of the embryos to be determined by visualization of *Hex* expression in the anterior visceral endoderm [88]. WISHs were carried out with 5′-Digoxigenin labeled Locked Nucleic Acid modified oligonucleotides (Exiqon, Denmark) as described previously [89]. For cryosectioning, embryos were embedded in OCT (Cell Path, UK) and sectioned at 10 µm using a Leica CM 1950 cryostat. Sections were mounted on slides with Vectamount (Vector Laboratories, USA).

### Cloning

Pri-miRNA luciferase assay constructs were generated by PCR-amplification of 1–1.5kb regions of the pri-miRNA genes from mouse genomic DNA, containing the putative ASE sites, and insertion of the PCR products into the multiple cloning site of the pGL3-promoter vector (Promega, USA). Mutations in the putative ASE sites of the pri-miR-341∼3072 miR-ASE construct were introduced by site-directed mutagenesis in PCR. Primer sequences are shown in Table S5.

### Luciferase Assays

ES cells were weaned from feeder cells before seeding onto 96-well plates and were maintained in 20% FBS with 2×LIF. The following day, cells were incubated with a total of 200ng plasmid DNA with experimental firefly luciferase and control renilla luciferase in a 1∶80 ratio using Lipofectamine 2000 (Invitrogen, UK) for 4–6 hrs, according to the instructions of the manufacturer. After the transfection, cells were treated overnight with 60 µM SB-431542 (SB), followed by 5 hrs treatment with either 20ng/ml Activin A or 60 µM SB. Cells were assayed using the Dual-Luciferase Reporter Assay System (Promega, USA). Luciferase activity of the experimental firefly luciferase plasmids was normalized to the control renilla plasmid. The relative luciferase activity of each construct after Activin treatment was compared to that of the SB-treated control.

### Sequence Analysis

The genomic coordinates of the candidate pri-miRNA genes were obtained from predictions described by Marson and colleagues [28]. Putative poly(A) sites were identified using the dnafsminer tooI [90], searching for 4 hexamer sequences (AATAAA, ATTAAA, AGTAAA, and TATAAA) with a cutoff score of 0.6.

In order to identify potential ASE and CAGA boxes, 10kb regions up- and downstream of the predicted pri-miRNA transcriptional start sites were searched with Fuzznuc, a program of the EMBOSS-MS software [91]. Potential ASE binding sites and CAGA boxes were identified by searching for the consensus binding sequences AATMMACA (M = C or A) and AGAC, respectively, in both orientations. The species conservation of the putative ASE sites was analyzed using the UCSC genome browser [92].

### miRNA Target Prediction

For computational miRNA target site predictions, Diana microT version 3.0 (diana.cslab.ece.ntua.gr/microT) [58] and microCosm version 5 (miRBase Targets database) were used (http://www.ebi.ac.uk/enright-srv/microcosm/htdocs/targets/v5/) [59], [60].

### Gene Function Annotation

The annotated information on each gene was compiled from the Mouse Genome Database (http://www.informatics.jax.org/) [93], Entrez Gene (www.ncbi.nlm.nih.gov/gene) [94] and Gene Ontology (http://www.geneontology.org/) [95]. Synonyms for each gene are shown in parentheses in the first column. Gene Ontology information showing known or predicted functions was obtained from all three databases and references therein. Gene expression in the different tissues and at different stages was curated from cDNA source data provided by the Mouse Genome Database.

## Supporting Information

Table S1 **Identification of Smad2/3 inducible miRNAs by small RNA deep-sequencing in TAG1 ES cells.** miRNAs displaying a significant p-Smad2/3 activation profile and a high abundance, also shown in Table 1.
(XLSX)

Table S2 **Response of the miRNAs of the putative pri-miR-341∼3072 cluster to changes in the p-Smad2/3 activation profile as detected by short RNA deep-sequencing.** ND = Not Detected.
(XLSX)

Table S3 **Expression and functional annotation of p-Smad2/3 repressed genes.** -There is no information available in the databases.
(DOCX)

Table S4 **Real-time RT-qPCR primer sequences.** For pri-miRNA RT-qPCR experiments in embryos, the above reverse primers were used for the reverse transcription reactions prior to amplification by qPCR.
(DOCX)

Table S5 **Primers used for generating pri-miRNA ASE Luciferase constructs.** Pri-miR-341∼3072 ASE(B) MUT construct was generated by overlap extension PCR of 3 amplicons as indicated by primer labels. Nucleotides introduced for mutagenesis are shown underlined. The insert for the WT pri-miR-341∼3072 ASE(B) construct was amplified using pri-miR-341∼3072 ASE(B) Fwd 1 and Rev 3 primer pair.
(DOCX)
