# Supplementary material for: TGF-β/Smad2/3 Signaling Directly Regulates Several miRNAs in Mouse ES Cells and Early Embryos
Source: PLoS One. 2013 Jan 30;8(1):e55186. doi: 10.1371/journal.pone.0055186 (PMC3559380; doi:10.1371/journal.pone.0055186)
Supplement: Table S3 — Expression and functional annotation of p-Smad2/3 repressed genes. -There is no information available in the databases. (DOCX) [file pone.0055186.s003.docx]

**­­­­**

**Table S3. Expression and functional annotation of p-Smad2/3 repressed genes**

| **Gene symbol (synonyms)** | **Gene name** | **Ontology** | **Component** | **Expression** |
| --- | --- | --- | --- | --- |
| Nceh1 (Aadacl1, CPO-BP, B230106I24Rik, mKIAA1363) | Arylacetamide deacetylase-like 1 | Lipid catabolic process, metabolic processes, protein dephosphorylation, carboxylesterase activity, hydrolase activity, phosphate ion binding | Endoplasmic reticulum, membrane, integral to membrane, intracellular membrane-bounded organelle | Embryonic (nervous system, sensory organs, teeth) |
| Btbd11(6330404E16Rik) | BTB (POZ) domain containing 11 | Protein heterodimerization binding | Membrane, integral to membrane | Embryonic (nervous system, head, thymus primordium, renal and urinary system, reproductive system) |
| Gm10664 | Predicted gene 10664 | - | - | Embryonic (early embryo, extraembryonic component, alimentary system, nervous system, cardiovascular system, liver and biliary system) Post-natal (alimentary system, liver and biliary system, renal and urinary system) |
| Meg3 (Gtl2, 2900016C05Rik, 3110050O07Rik, 6330408G06Rik, D12Bwg1266e) | Maternally expressed 3 | Determination of adult life span, *in utero* embryonic development, liver development, lung alveolus development, multicellular organism growth, post-embryonic development, DNA methylation, genetic imprinting, skeletal muscle tissue development | - | Widely expressed |
| Hexa (Hex-1) | Hexosaminidase A | Adult walking behavior, cell morphogenesis involved in neuron differentiation, lipid storage, locomotory behaviour, lysosome organization, catabolic and metabolic processes, myelination, neuromuscular processes controlling balance and posture, sensory perception of sound, sexual reproduction, skeletal system development, beta-N-acetylhexosaminidase activity, catalytic activity, cation binding, hydrolase activity acting on glycosyl bonds and hydrolyzing O-glycosol compounds | Lysosome, membrane | Embryonic (widely expressed) Post-natal (nervous system) |
| Lnpep (2010309L07Rik, 4732490P18Rik, gp160, IRAP, vp165) | Leucyl/cystinyl aminopeptidase | Protein catabolic process, proteolysis, aminopeptidase activity, hydrolase activity, metal ion binding, metallopeptidase activity, peptidase activity, zinc ion binding | Membrane, perinuclear region of cytoplasm, plasma membrane, integral to membrane | Embryonic (head, skin) Post-natal (caecum, kidney, skin, intestine, spleen, testis, thymus, vagina) |
| Phf15 (Jade2, mKIAA0239, 1200017K05Rik) | PHD finger protein 15 | Histone acetylation, metal ion binding | Histone acetyltransferase complex | Embryonic (widely expressed) Post-natal (nervous system, sensory organs, head) |
| Rbm14 (1300007E16Rik, p16, PSP2) | RNA binding motif protein 14 | - | - | Embryonic (limbs, nervous system, sensory organs)  Post-natal (nervous system, sensory organs) |
| Stoml1 (1810015E19Rik, SLP-1, UNC-24, WPB72) | Stomatin-like 1 | Sterol binding | Integral to membrane, membrane | Widely expressed |
| Sub1 (P15, Pc4, Rpo2tc1) | SUB1 homolog (S. cerevisiae) | Regulation of transcription from RNA polymerase II promoter, DNA-dependent regulation of transcription, DNA binding, transcription coactivator activity, single-stranded DNA binding | Nucleolus, nucleus, transcription factor complex | Embryonic (widely expressed) Post-natal (nervous system, sensory organs) |
| Vim | Vimentin | Astrocyte development, Bergmann glial cell differentiation, intermediate filament-based process, lens fiber cell development, negative regulation of neuron projection development, protein binding, structural constituent of eye lens, structural molecule activity | Cell leading edge, cell projection, cytoplasm, cytoskeleton, intermediate filament | Widely expressed |
| -There is no information available in the databases | | | | |
