# Supplementary material for: TGF-β/Smad2/3 Signaling Directly Regulates Several miRNAs in Mouse ES Cells and Early Embryos
Source: PLoS One. 2013 Jan 30;8(1):e55186. doi: 10.1371/journal.pone.0055186 (PMC3559380; doi:10.1371/journal.pone.0055186)
Supplement: Table S4 — Real-time RT-qPCR primer sequences. For pri-miRNA RT-qPCR experiments in embryos, the above reverse primers were used for the reverse transcription reactions prior to amplification by qPCR. (DOCX) [file pone.0055186.s004.docx]

**Table S4. Real-time RT-qPCR primer sequences**

| **Gene** | **Fwd primer (5’- 3’)** | **Rev primer (5’-3’)** |
| --- | --- | --- |
| Pri-mir-7a-1 | TGGCCTAGTTCTGTGTGGAA | GTCCTGTAGAGGTGGCCTGT |
| Pri-mir-7a-2 | GAGACTCTAGGGAACTGTATGAGC | AATCACTAGTCTTCCAAACGG |
| Pri-mir-17 | CAGTGAGGGCACTTGTAGCA | GACTGACCGACGACTGGAC |
| Pri-mir-106b~25 | TTGTCTCGGTCTGACAGTGC | GCAAGCCACATTTGCCTACT |
| Pri-mir-29a | GCACATGACCTCTTGTGACC | AACCGATTTCAGATGGTGCT |
| Pri-mir-130a | AAATGAGGACGAGGGACG | AACATTGCACTGCTCGGT |
| Pri-mir-185 | ATCTGGGCTTAGGTAAAGG | AGGGGACCATCAGGAACT |
| Pri-mir-181c/d | GCCAAACAACACCATCAGG | CCAGACACCAGTCCCATTTC |
| Pri-mir-290~295 | AGACTGAGTATAGCGAAAGGCTA | AGCACTTTCTCATTCAGGC |
| Pri-mir-341~3072 | CGTGGTGGATTCGCTTTACT | CTTTCTGCAAGTTGCCATCA |
| Pri-mir-744 | ACTGCCAAGGTGAGTTCA | CCGTCAGTAAGACTGCTGTTAG |
| Gapdh | AACTTTGGCATTGTGGAAGG | ACACATTTGGGGGTAGGAACA |
| Nodal | GAGTTTCATCCTACCAACCA | TGCCATTGTCCACATAAAGC |
| For pri-miRNA RT-qPCR experiments in embryos, the above reverse primers were used for the reverse transcription reactions prior to amplification by qPCR | | |
