# Supplementary material for: TGF-β/Smad2/3 Signaling Directly Regulates Several miRNAs in Mouse ES Cells and Early Embryos
Source: PLoS One. 2013 Jan 30;8(1):e55186. doi: 10.1371/journal.pone.0055186 (PMC3559380; doi:10.1371/journal.pone.0055186)
Supplement: Table S5 — Primers used for generating pri-miRNA ASE Luciferase constructs. Pri-miR-341∼3072 ASE(B) MUT construct was generated by overlap extension PCR of 3 amplicons as indicated by primer labels. Nucleotides introduced for mutagenesis are shown underlined. The insert for the WT pri-miR-341∼3072 ASE(B) construct was amplified using pri-miR-341∼3072 ASE(B) Fwd 1 and Rev 3 primer pair. (DOCX) [file pone.0055186.s005.docx]

**Table S5. Primers used for generating pri-miRNA ASE Luciferase constructs**

| **Primer** | **Sequence (5’-3’)** |
| --- | --- |
| Pri-miR-181c/d ASE(A) Fwd MluI | AAAAAAACGCGTGGTAACAGCCTGGGTTTGAGGG |
| Pri-miR-181c/d ASE(A) Rev BglII | AAAAAAAGATCTCCCTCGTACTCCACGCTGCAGC |
| Pri-miR-181c/d ASE(B) Fwd MluI | AAAAAAACGCGTGCCTGAGCTTCAGGGTAGTAAGGAAGT |
| Pri-miR-181c/d ASE(B) Rev BglII | AAAAAAAGATCTGCCCTATCTCTGTGTCCTATGTCCCA |
| Pri-miR-341~3072 ASE(A) Fwd MluI | AAAAAAACGCGTCTGTTCTCCCAGGATTGCCTGG |
| Pri-miR-341~3072 ASE(A) Rev BglII | AAAAAAAGATCTCGCAGATGAACTTGGGGATGAA |
| Pri-miR-341~3072 ASE(B) Fwd 1 MluI | AAAAAAACGCGTCCTTAGCATAATGTCCACGTTGGG |
| Pri-miR-341~3072 ASE(B) MUT Rev 1 | GTGGGTTTTTGTATTGATTAAGATCATTAAATCA |
| Pri-miR-341~3072 ASE(B) MUT Fwd 2 | TAATCAATACAAAAACCCACAGTT |
| Pri-miR-341~3072 ASE(B) MUT Rev 2 | TGGTGTGCTGTTTACTGCG |
| Pri-miR-341~3072 ASE(B) MUT Fwd 3 | ACGCAGTAAACAGCACACCAAATGCTCTTACCTT |
| Pri-miR-341~3072 ASE(B) Rev 3 BglII | AAAAAAAGATCTCCTCCCGTGTATACAGAATCCGAGG |
| Pri-miR-341~3072 ASE(C) Fwd MluI | AAAAAAACGCGTCATCAAGTGCTTCTCTGAAGACGTGG |
| Pri-miR-341~3072 ASE(C) Rev BglII | AAAAAAAGATCTCCTCCCGTGTATACAGAATCCGAGG |
| Pri-miR-341~3072 ASE(B) MUT construct was generated by overlap extension PCR of 3 amplicons as indicated by primer labels. Nucleotides introduced for mutagenesis are shown underlined. The insert for the WT pri-miR-341~3072 ASE(B) construct was amplified using pri-miR-341~3072 ASE(B) Fwd 1 and Rev 3 primer pair | |
